# Bacteriophage Applications for Food Production and Processing

**DOI:** 10.3390/v10040205

**Published:** 2018-04-19

**Authors:** Zachary D. Moye, Joelle Woolston, Alexander Sulakvelidze

**Affiliations:** Intralytix, Inc., The Columbus Center, 701 E. Pratt Street, Baltimore, MD 21202, USA; jwoolston@intralytix.com (J.W.); asulakvelidze@intralytix.com (A.S.)

**Keywords:** bacteriophages, phages, food safety, foodborne illness

## Abstract

Foodborne illnesses remain a major cause of hospitalization and death worldwide despite many advances in food sanitation techniques and pathogen surveillance. Traditional antimicrobial methods, such as pasteurization, high pressure processing, irradiation, and chemical disinfectants are capable of reducing microbial populations in foods to varying degrees, but they also have considerable drawbacks, such as a large initial investment, potential damage to processing equipment due to their corrosive nature, and a deleterious impact on organoleptic qualities (and possibly the nutritional value) of foods. Perhaps most importantly, these decontamination strategies kill indiscriminately, including many—often beneficial—bacteria that are naturally present in foods. One promising technique that addresses several of these shortcomings is bacteriophage biocontrol, a green and natural method that uses lytic bacteriophages isolated from the environment to specifically target pathogenic bacteria and eliminate them from (or significantly reduce their levels in) foods. Since the initial conception of using bacteriophages on foods, a substantial number of research reports have described the use of bacteriophage biocontrol to target a variety of bacterial pathogens in various foods, ranging from ready-to-eat deli meats to fresh fruits and vegetables, and the number of commercially available products containing bacteriophages approved for use in food safety applications has also been steadily increasing. Though some challenges remain, bacteriophage biocontrol is increasingly recognized as an attractive modality in our arsenal of tools for safely and naturally eliminating pathogenic bacteria from foods.

## 1. Introduction

From leaves of lettuce and cheddar cheese in a Cobb salad to frozen pre-cooked meals, the foods we eat remain under constant threat of contamination by microbial pathogens, which can subsequently be transmitted to the consumer. Recently, the Foodborne Disease Burden Epidemiology Reference Group (FERG) was established by the World Health Organization (WHO) to monitor foodborne illness across the world. FERG monitored the 31 foodborne pathogens that caused the highest morbidity and mortality in humans. In their most recent (2015) estimate of the global burden of foodborne illness, FERG approximated that 600 million foodborne infections occurred in 2010, resulting in over 400,000 deaths. Of the top five microorganisms causing foodborne illness, four were bacteria: *Escherichia coli* (~111 million), *Campylobacter* spp. (~96 million), non-typhoid *Salmonella enterica* (~78 million), and *Shigella* spp. (~51 million), with estimates for the number of foodborne-related deaths caused by these bacteria ranging from ~15,000 for *Shigella* spp. to ~63,000 for *E. coli* [1]. Strikingly, children under five years old were disproportionally impacted; they account for 40% of deaths while representing just 9% of the world population [1]. These foodborne illnesses are also a tremendous drain on the economy of nations; for example, in the United States the average incident is estimated to cost ~$1500/person, with the total annual estimated cost of these foodborne diseases reaching over $75 billion [2]. 

Several approaches are used to help improve the safety of our foods. Heat pasteurization is commonly used to reduce bacterial numbers in liquids and dairy items, most notably milk. However, pasteurization is not suitable for many fresh food items, as the process results in the items being cooked. Another method used to reduce pathogens in foods is High Pressure Processing (HPP) which exposes foods to high pressure to inactivate microbes. This technique has been successfully used on liquid products and pre-cooked meals, meant to be frozen; however, as with heat pasteurization, it is generally not used with fresh meats and produce, as it can affect the appearance (color) and/or nutritional content of these products [3,4]. Irradiation is also an effective means for reducing the burden of pathogenic organisms in foods. However, irradiation can deleteriously affect the organoleptic qualities of foods; in addition, customer acceptance of this method is low and is compounded by a labelling requirement for many food items treated with radiation [5,6]. Finally, chemical sanitizers, such as chlorine and peracetic acid (PAA), are commonly utilized to reduce microbial contaminants of many fresh fruits and vegetables as well as Ready-To-Eat (RTE) food products [7,8]. While they are, in general, effective, many of these chemicals are corrosive and can damage food processing equipment. Chemical sanitizers can also deleteriously affect the environment (i.e., not environmentally-friendly) and, with the current trends toward chemical-free, organic foods, consumer acceptance of chemical additives in foods (particularly in fresh produce) is declining rapidly. One common downside shared by all of these techniques is that they kill microbes indiscriminately; in other words, both the pathogenic as well as potentially advantageous normal flora bacteria are targeted equally. Additionally, even with the variety of methods available, foodborne outbreaks still occur relatively frequently. These factors combined illustrate the need for a targeted antimicrobial approach, one that can be used alone or in combination with the techniques described above, to establish additional barriers in a multi-hurdle approach to preventing foodborne bacterial pathogens from reaching consumers. One such technique is the use of lytic bacteriophages for targeting specific foodborne bacteria in our foods, without deleteriously impacting their normal—and often beneficial—microflora. This approach is termed “bacteriophage biocontrol” or “phage biocontrol”.

Phage biocontrol is increasingly accepted as a natural and green technology, effective at specifically targeting bacterial pathogens in various foods, in order to safeguard the food chain (Table 1). Bacteriophages were first identified by Felix d’Herelle in 1917, and the usefulness of these “bacteria eaters” for combating bacterial diseases was quickly exploited [9]. In the context of food safety, bacteriophages address many of the concerns voiced by consumers. For example, because of the specificity of bacteriophages, phage biocontrol offers a unique opportunity to target pathogenic bacteria in foods without disturbing the normal microflora of foods. Of note, the United States Army recently initiated a project (W911QY-18-C-0010) to further elucidate the impact of phage application versus traditional chemical antimicrobials on the normal microbiota of fresh produce and how these interventions may impact the nutritional value of foods. Also, phage biocontrol is arguably the most environmentally-friendly antimicrobial intervention available today. Most, if not all, currently-available commercial phage biocontrol products contain natural phages, i.e., phages isolated from the environment, that are not genetically modified. Many of these preparations also do not contain any additives or preservatives; they are typically water-based solutions consisting of purified phages and low levels of salts. Several phage preparations on the market are also certified Kosher and Halal and are available for use in organic foods (OMRI-listed in USA; SKAL in EU) (Table 2). Although there is limited testing, work conducted by our group suggests that bacteriophages do not alter the organoleptic (i.e., sensory) properties of foods [10]. Finally, compared to other food safety interventions, the cost of applying bacteriophages is relatively low and is typically in the range of 1–4 cents per pound of food treated; whereas HPP treatment and irradiation typically cost 10–30 cents per pound [11]. It is important to note that these figures represent the cost of each intervention alone, and do not account for situations where a multi-hurdle approach may be required for food safety purposes (e.g., foods are feared to be contaminated by more than one foodborne pathogen) or for considerations apart from food safety (e.g., food spoilage which is typically caused by multiple different microorganisms). 

The biological properties of lytic bacteriophages and other qualities of commercial phage biocontrol products as explained above make phage biocontrol a very attractive modality for further improving the safety of our foods, and an increasing number of companies worldwide are engaging in their development and commercialization [12] (Table 2). However, phage biocontrol does have its limitations and drawbacks. For example, phage preparations require refrigerated storage (typically 2–8 °C), and if used in conjunction with chemical sanitizers, may need to be applied separately, as harsh chemicals can also inactivate the phage particles and render phage biocontrol less effective. Also, because of their high natural specificity, phage preparations can effectively address targeted pathogens in foods, but if food items happen to be contaminated with two or more foodborne bacterial pathogens, a phage preparation targeted against a single pathogen will not be effective in removing non-targeted pathogenic bacteria from foods. As a final consideration, care must be taken to use lytic phages and exclude temperate phages from bacteriophage preparations. Temperate phages are typically less effective than lytic phages at killing their bacterial hosts. Moreover, temperate phages are capable of integrating their DNA into the bacterial chromosome, and therefore, they can potentially promote the transfer of virulence genes or other undesirable genes (e.g., antibiotic-resistance encoding genes) among bacterial strains, which could lead to the emergence of new pathogenic strains. The risk of such emergence is significantly lower when lytic phages are utilized.

This review is focused on applications of wild type bacteriophages for improving the safety of foods. We do not discuss other possible phage-related methods such as, for example, the use of phage endolysins for targeting foodborne pathogens, or using bacteriophages to manage food spoilage. Those topics have been discussed by other authors previously and respective reviews are available [13,14]. In the context of food safety applications, wild type lytic bacteriophages can be used both pre-harvest (e.g., in live animals, administered via animal feed or spray-applied prior to slaughter) and/or post-harvest (e.g., applied directly to food surfaces, either via direct spraying, via packaging materials, or by some other means) to reduce contamination by pathogenic bacteria [12,15]. Bacteriophage biocontrol could also be a means to disinfect surfaces used in the production and processing of foods [16,17]. In previous reviews [12,14,18,19], we and others have assembled a general overview of the industries and products where bacteriophages are used in food safety applications. Here, we provide an updated review (and an extended summary table) describing studies where bacteriophages have been applied to predominantly post-harvest foods, particularly meats, fresh produce and RTE foods (Table 1). In the next section, we review selected studies from the last five years where bacteriophage biocontrol was used to combat four major foodborne pathogens. Finally, we also discuss the regulation of bacteriophages for food safety applications and some of the challenges of phage biocontrol. 

## 2. Phage Biocontrol for Targeting Common Foodborne Bacterial Pathogens

### 2.1. Listeria monocytogenes

*Listeria monocytogenes* is a rod-shaped, Gram-positive, facultative anaerobe. Consumption of foods contaminated with *L. monocytogenes* causes a range of symptoms in humans such as initial flu-like or gastrointestinal symptoms which, in some cases, progress to encephalitis or cervical symptoms, and possibly stillbirth in pregnant mothers. It was estimated that in 2010, global cases of foodborne infection with *L. monocytogenes* exceeded 14,000 and resulted in more than 3000 deaths [1]. *L. monocytogenes* is able to survive and grow at refrigerated temperatures (2–8 °C) commonly used during the distribution and storage of many foods; therefore, the detection and elimination of *L. monocytogenes* is critically important to ensuring the safety of the food chain, especially in RTE foods. In this context, the application of bacteriophages to assorted foods (including RTE foods) has been shown, by several investigators, to be effective at reducing contamination with *L. monocytogenes* (Table 1). For example, a commercial monophage preparation (i.e., phage preparation consisting of one single phage) targeting *Listeria* was reported to be effective in reducing the levels of *L. monocytogenes* in sliced ham, and to be superior to nisin and sodium lactate, when compared at the storage abuse temperature of 6–8 °C [47]. A similar study by Chibeu and colleagues (2013) demonstrated that the same monophage preparation was also able to reduce *L. monocytogenes* on the surface of other deli meats [44]. The meats (cooked sliced turkey and roast beef) were stored at 4 °C and the abuse temperature of 10 °C. The *Listeria*-specific phage was effective against *L. monocytogenes* when used alone, and it enhanced the effectiveness of other antimicrobials when used together with sodium diacetate or potassium lactate. All these studies utilized a single phage preparation. A phage cocktail prepared with multiple bacteriophages compared to a single phage preparation may be superior, both in terms of providing broader coverage of the target species and of reducing the risk of resistant bacteria emerging. One such commercially available six-phage cocktail targeting *L. monocytogenes* has been tested on a number of foods experimentally contaminated with *L. monocytogenes*, including lettuce, hard pasteurized cheese, smoked salmon, and Gala apple slices; application of this bacteriophage cocktail reduced *L. monocytogenes* levels in all these foods by ~0.7–1.1 logs [10]. The same study examined the application of the *L. monocytogenes*-specific cocktail on prepackaged, frozen meals. The meals were experimentally contaminated with *L. monocytogenes,* treated with the phage cocktail, and subjected to freezing and thawing cycles. The results showed a 2.2 log reduction of *L. monocytogenes*, which suggests that phage biocontrol can be an effective means to control *L. monocytogenes* in foods under “storage abuse” conditions when the frozen meals are intentionally or unintentionally thawed multiple times during their storage [10]. 

In many of the above-reviewed studies, despite the initial significant reduction in *L. monocytogenes* levels in the foods, the targeted bacterial populations were not completely eradicated, and viable *L. monocytogenes* cells could still be recovered, albeit in much lower numbers. However, the bacteriophage preparations were still effective against randomly selected colonies of the recovered bacteria, suggesting that phage-resistance was not the primary reason for the incomplete eradication of *L. monocytogenes* [23,37,44]. There could be several possible explanations for this observation. For example, the *L. monocytogenes* cells could be exhibiting temporal resistance to phage infection, as reported previously [70,71]. Another possible explanation is that the phages did not come into direct contact with some *L. monocytogenes* cells after the phages were sprayed onto the foods (e.g., due to using an insufficient volume of spray, particularly on foods with complex topography), which resulted in those bacterial cells not being lysed by phages. In this latter scenario, using larger spray volumes, fine (mist-like) sprays, rotating/tumbling foods during phage application, and otherwise ensuring thorough surface coverage with phages may help enhance the effectiveness of phage biocontrol.

### 2.2. Salmonella spp.

The non-typhoid serotypes of *Salmonella enterica* account for many incidents of gastroenteritis worldwide each year. The disease caused by these Gram-negative, rod-shaped bacteria is often self-limiting, with symptoms typically including stomach cramps, fever, nausea and diarrhea. However, life-threatening instances can occur in cases of dehydration and when the bacteria invade beyond the gastrointestinal tract. Estimates indicate that globally over 78 million cases of foodborne infection were caused by non-typhoid *Salmonella* in 2010, leading to almost 60,000 deaths [1]. During the processing and packaging of foods, *Salmonella*, as well as other pathogens, can adhere to the surfaces where food is prepared, leaving them contaminated. These factors place RTE foods, such as fresh fruits and vegetables that are not cooked before eating, at a particularly high risk for transmitting bacterial pathogens and causing food poisoning.

At least two FDA-cleared *Salmonella*-targeting phage preparations are currently on the market (Table 2). Several publications are available describing their applications (and that of other noncommercial phage preparations) in various foods. Brief summaries of those studies are given in Table 1. One study is of particular interest, as it demonstrates an example of how phage-resistance could be managed if and when it hinders the efficacy of a bacteriophage preparation. In that study, a GRAS-listed (Generally Recognized as Safe) six-phage cocktail targeting *Salmonella* was examined for its ability to reduce the levels of *Salmonella* on surfaces mimicking those commonly found in food processing establishments, e.g., stainless steel and glass [16]. Initial studies demonstrated that the *Salmonella*-specific bacteriophage cocktail significantly reduced the population of susceptible *Salmonella* strains on all surfaces examined by ~2–4 logs; at the same time, it was ineffective in reducing the levels of another strain of *Salmonella* (*Salmonella* Paratyphi B S661) that was resistant to the phage cocktail *in vitro* [16]. However, when the phage cocktail was adjusted to include phages specifically targeting this resistant strain, the updated preparation showed a significant reduction (~2 logs) of *S.* Paratyphi B S661 from the surfaces, while also maintaining effectiveness against the previously susceptible isolates [16]. This study provides compelling evidence that phage cocktails can easily be modified to target specific bacterial strains, e.g., if phage-resistant mutants emerge, or to specifically target the problem strains prevalent in particular food-manufacturing facilities.

In addition to their usefulness in decontaminating food preparation surfaces, bacteriophage cocktails have also been effective at eliminating *Salmonella* directly from the foods. For example, the same *Salmonella*-specific cocktail discussed above reduced the levels of *Salmonella* on experimentally contaminated chicken parts when applied alone, and this effect was enhanced when the phage was applied in combination with conventional chemical sanitizers [59]. In the case of chicken breast fillets, the bacteriophage cocktail significantly reduced the numbers of a mixture of *Salmonella* species when applied to the surface of the fillets or when the fillets were dipped into a vessel containing the phage solution [60]. Furthermore, this phage cocktail significantly reduced the number of *Salmonella* when the fillets were stored under aerobic or modified atmospheric conditions [60]. This latter finding may have direct practical implications as food manufacturers often use modified atmospheric conditions to discourage growth of bacteria and increase the shelf life of foods. Another study found that a single phage, SJ2, significantly reduced the amount of *Salmonella* in liquid egg and ground pork, and this reduction was more pronounced at higher temperatures [62]. The authors screened remaining *Salmonella* colonies for resistance; while there was no difference in the number of resistant clones from phage treated and untreated samples of ground pork, there was a significantly higher number of resistant clones found in the phage treated samples of egg liquid [62]. The authors suggested both the food matrix (solid versus liquid) and the differences in the microbiome of the two foods could have contributed to this difference in the number of resistant *Salmonella* isolates [62].

Foodborne illnesses caused by non-typhoid serotypes of *Salmonella* are also a health risk for companion animals (e.g., dogs and cats), and the close association of these animals with their owners raises the possibility of illness in humans. Indeed, human *Salmonella* outbreaks have been associated with contaminated dry cat and dog food, and approximately one third of commercial raw and natural pet foods sampled have been found to contain *Salmonella* [72,73]. In an effort to address this health risk, phage biocontrol has recently been examined as a technique to reduce or eliminate *Salmonella* in pet foods. The six-phage *Salmonella*-specific cocktail discussed above was found to reduce the levels of *Salmonella* in experimentally contaminated dry dog food by 1 log [74]; when cats and dogs were fed dry kibble treated with the same phage cocktail, it appeared to be safe and did not noticeably impact any of the major health metrics recorded for either of the animals [61]. 

An alternative to dry pet chow that is gaining increasing popularity is raw pet food. These pet meals consist of meats, such as chicken, duck or tuna, combined with vegetables, including lettuce, blueberries and broccoli, which are sold and served raw [61]. Raw pet foods are gaining increased popularity due to their superb nutritional values; at the same time, because they are uncooked, there is a heightened possibility of foodborne pathogens being present in them, which can be transferred to pets as well as to unsuspecting consumers during the feeding process. At least one report was recently published in which the authors examined the value of using phages to control *Salmonella* in raw pet food ingredients. Reductions of bacterial contamination ranged from 0.4 log to 1.1 logs, the efficacy was concentration-dependent, and the largest reduction was achieved when high doses of the bacteriophage preparation were used [61] (Table 1).

### 2.3. Escherichia coli

Many strains of the Gram-negative, rod-shaped bacteria *Escherichia coli* are naturally found in the human gut and are beneficial for our health and wellbeing; for example, they aid in the digestion of food and maintenance of a robust immune system. However, some *E. coli* strains can and do cause illnesses in humans. For example, the Shiga toxin producing *E. coli* serotype O157:H7, which is sometimes found in contaminated water or foods, especially beef, can invade the human gastrointestinal tract and trigger disease, with symptoms including abdominal cramping and hemorrhagic diarrhea. These infections are typically self-limiting in immunocompetent individuals but can potentially be life-threatening in very young or old patients. It has been estimated that globally more than one million cases of foodborne illness and over one hundred deaths could be attributed to Shiga toxin-producing *E. coli*, including the O157:H7 serotype [1]. 

Recent work has demonstrated that *E. coli*-specific phage preparations were effective when used to treat fresh vegetables [75] and both Ultra-High-Temperature (UHT) treated and raw milk contaminated with *E. coli* [33]. In the first study, the levels of *E. coli* O157:H7 on green pepper slices and spinach leaves were reduced by a single phage by ~1–4 logs, and the initial reduction was maintained at 4 °C while some regrowth was seen at 25 °C. In the second study, the levels of *E. coli* were reduced to undetectable levels in both UHT and raw milk when a cocktail of two or three phages was used. Of note, in all samples treated with the three-phage preparation, this reduction was maintained over storage at both 4 and 25 °C; in contrast, there was regrowth of the *E. coli* strain in the samples treated with the two-phage cocktail. While the underlying reasons are not fully understood, it is possible that the three-phage cocktail provided better management of resistance versus a two-phage cocktail, and the enhanced efficacy of multi-phage cocktails has been demonstrated for other phage preparations previously [76]. Although the underlying reasons for this phenomenon have not been rigorously determined, it is possible that having multiple phages in a phage cocktail reduces the risk of the emergence of phage-resistant mutants, because multiple mutations would be required to render a given bacterial cell resistant to not one, but multiple phages in the cocktail, assuming the phages target distinct cellular structures. This concept is essentially the same as the multi-hurdle approach, which proposes using a combination of antibacterial strategies to discourage the development of bacterial resistance [77]. These and some additional studies using *E. coli*-specific phages in food safety applications are briefly summarized in Table 1.

### 2.4. Shigella spp.

Species of the Gram-negative, rod-shaped bacterial genus *Shigella* cause a self-limiting gastrointestinal infection with symptoms including hemorrhagic diarrhea and stomach pain. Globally, the incidence of foodborne infection caused by *Shigella* species in 2010 was recently estimated to be over 50 million, resulting in over 15,000 deaths [1]. The vast majority of these infections occurred in developing countries, with the highest number of the infections and death occurring in children under the age of 5 [1,78]. 

Only one FDA-cleared food safety phage preparation is currently available to target *Shigella* spp. [66,69]. This five-phage cocktail was granted the GRAS status in 2017 (GRN 672) (Table 2), and it was shown to reduce the levels of *Shigella* by approximately 1 log in a variety of foods, including melons, lettuce, yogurt, deli corned beef, smoked salmon, and chicken breast meat [66]. In another study, the same *Shigella*-specific bacteriophage cocktail was used to compare the safety and efficacy of phage administration to antibiotic treatment in mice challenged with a *Shigella sonnei* strain [69]. This study demonstrated that, while the *Shigella* specific bacteriophage cocktail was as effective as a standard antibiotic treatment at reducing the bacterial load in mice, treatment with the antibiotic significantly altered the diversity of the mouse intestinal community, while the phage treatment did not i.e., phage administration had a much milder impact on the normal gut microbiota of mice, compared to the antibiotic treatment [69]. The authors did not observe any deleterious side effects in the mice after phage administration, that is, the phage did not alter the composition of the blood or urine of the mice, nor did it have any detrimental effect on the morbidity or mortality, weight or any other physiological parameters of the animals [69]. Although not directly relevant to food safety applications, the study did suggest that these bacteriophages, when administered orally (mimicking a scenario when they would be consumed when eating foods treated with them) did not deleteriously impact the normal gut microflora (in contrast to antibiotics) and triggered no side effects in any of the animals examined.

### 2.5. Campylobacter jejuni

*Campylobacter* spp., Gram-negative, curved rod-shaped bacteria, are major foodborne pathogens of humans, causing gastrointestinal symptoms that can include stomach pain, fever and diarrhea. It a recent (2015) report, FERG estimated that in 2010, the global cases of foodborne illness caused by *Campylobacter* spp. exceed 95 million and resulted in over 21,000 deaths [1]. The intestinal microflora of many fowl and other livestock animals include species of *Campylobacter*. Additionally, though the route of entry is not fully understood, *Campylobacter* can frequently be isolated from both the surface of and internally within chicken livers. Zoonotic infections commonly occur in humans when contaminated animal products, such as meats, are handled or consumed. Thus, humans are at an elevated risk for *Campylobacter* infection when minimally cooked preparations, e.g., pâté, are prepared. 

Several *Campylobacter* bacteriophages have been isolated from chickens, including the fecal matter as well as the surface and internal tissues of chicken livers, and some of them have been examined for their ability to reduce contamination of various foods by *Campylobacter* [79,80,81,82]. For example, Hammerl and colleagues [80] used the phages as a pre-harvest treatment, and showed significant reduction (~3 logs) in *Campylobacter* fecal counts when 20-day-old chickens were treated with two phages in successive application (a Group III phage, then a Group II phage). Interestingly, dosing of the Group III phage alone or in conjunction with another Group III phage was not effective, suggesting that a combination of different phages (Group II and III) was required for optimal efficacy. The isolation of *Campylobacter*-specific phages has historically been done on a limited number of *Campylobacter* isolates, with many studies utilizing just one *C. jejuni* NCTC 12662 isolate as a host strain for phage isolation. Phages isolated using that one strain are almost exclusively Group III phages that target a particular receptor, the capsular polysaccharide [83]. In contrast, phages isolated on *C. jejuni* RM1221 are typically Group II phages that utilize the flagella as a route of entry [83]. As the above study suggests [80], phage cocktail consisting of phages that target different receptors could potentially lead to a broader target range and more effective cocktails.

## 3. Bacteriophage Preparations as Commercial Products

### 3.1. Regulation of Bacteriophage Preparations

In the last approximately 12 years, the number of regulatory approvals issued for bacteriophage preparations and their use for improving food safety has been steadily increasing (Table 2). In 2006, the first approval for a bacteriophage preparation to be used directly in the food supply was issued by the FDA for the *L. monocytogenes*-specific cocktail ListShield™ as a food additive (FDA does not “approve” any products, phage-based or otherwise; however, the term “approval” is commonly used to indicate obtaining FDA clearance to use products for their intended applications). Later that year, the FDA issued a no-objection letter for the *Listeria*-specific preparation Listex™ (currently PhageGuard Listex™) as a Generally Recognized as Safe (GRAS) substance. In recent years, a number of phage products (e.g., SalmoFresh™ and PhageGuard S™) have been granted GRAS designation by the FDA, and application for GRAS designation now appears to be the standard route of approval for phage products intended to treat post-harvest foods. As wild-type (i.e., not genetically modified) lytic bacteriophages are all natural and already inherently present in the food supply, the GRAS designation does seem to be an appropriate regulatory avenue for such preparations. In addition, the USDA has included several phage preparations in their issued guidelines for safe and suitable ingredients used in the production of meat, poultry, and egg products. For example, under FSIS Directive 7120.1, the application of phage to livestock animals prior to slaughter (e.g., *E. coli* O157:H7-targeted phages to the hides of cattle) and food (e.g., *Salmonella*-targeted phage onto poultry or meat) is permitted. These guideless were developed using specific phage preparations, but, in general, any phage product that meets the description in the directive may be considered to be compliant. Following the lead of regulators in the United States, several health agencies in countries around the world have issued approvals of phage products for use on foods; some examples include Israel, Canada, Switzerland, Australia, New Zealand, and the European Union (Table 2). 

### 3.2. Challenges for Bacteriophage Biocontrol 

As described in the previous sections, bacteriophage biocontrol is being increasingly used for targeting specific pathogenic bacteria in various foods, with a growing body of literature attesting to the utility of bacteriophages to reduce or eradicate their targeted pathogenic bacteria in foods. However, some challenges still remain before bacteriophage biocontrol is more widely accepted, including technical constraints and the general consumer acceptance of phage application on foods. Some of these challenges are briefly discussed below.

#### 3.2.1. Technical Challenges

Arguably the biggest technical challenge with phage biocontrol is its efficacy. One common observation in studies using bacteriophages on foods is that levels of contaminating bacteria drop initially, and very little or no further reduction in bacteria occurs afterwards [54,56]. In other words, phages can effectively reduce the levels of their targeted bacteria in foods, but they do not always eliminate them fully. Bacteriophage must come into contact with susceptible bacterial cells to lyse them. Given the nature of phage replication cycle (which starts with one phage infecting one bacterial cell and resulting in 100–200 progeny phages bursting from that cell at the end of each replication cycle; i.e., an exponential effect), one could expect that the reduction in bacterial cells will exponentially increase with more replication cycles, as more progeny phages are generated as the result of ongoing phage-mediated lysis of the targeted bacteria. However, several reports have suggested that the concentration of phages does not substantially increase after application to foods [43,44,45], strongly suggesting that “autodosing” (exponential increases in the population of phages due to repetitive lytic replication cycles) does not occur, at least under the conditions tested to date. It is likely that the progeny phages are unable to reach and invade additional bacteria in foods, especially in drier food matrixes, where passive movement of phages across food surfaces is limited due to the lack of moisture. In this context, it has been suggested that fewer phage particles may be required to significantly reduce bacterial contamination on moist food surfaces and in liquids compared to drier food matrices, presumably because of the increased “mobility” of phages in the presence of moisture (e.g., natural juices of some foods) [84]. One potential answer to this challenge is to use a phage solution with higher concentrations of phage particles, to increase the likelihood of phages coming into contact with their targeted bacteria upon application [17,21,36,66]; however, a more concentrated solution will be more expensive, to the point that it may be prohibitively expensive for food processors to implement. Another option is the use of larger spray volumes applied via fine mist sprays, to more efficiently disperse the phage particles across the surface of the food, increasing their likelihood of encountering a target bacterium, which could be especially important under circumstances when foodborne pathogens are present in very low concentrations or when the infective dose of the pathogen is extremely low. Proper application of bacteriophages onto foods to ensure thorough surface coverage and optimal efficacy is one of the main technical challenges for phage biocontrol, and it encompasses a range of issues from phage dosing (i.e., effective concentration of phage delivered in an optimal volume and how these could be verified in food processing facilities) to proper equipment (both to provide accurate dosing, as just mentioned, and appropriate mixing or tumbling during phage application to ensure that the entire surface of food is thoroughly treated with the phage solution).

An additional efficacy-related issue is that phage biocontrol typically reduces the levels of targeted bacteria by 1–3 logs (with rare exceptions: in one study, a reduction of *Listeria* of up to 5 logs was reported as a result of phage treatment [36]), and this is considerably lower than the up to 5 logs reduction reported for some other, more harsh interventions, e.g., irradiation. Although this may be more a perception problem than a real technical issue (since very few, if any, foods are contaminated with 5 logs of foodborne pathogens per gram), the lower reduction may be considered by the food industry to be inferior. Even when the targeted bacterium is not totally eliminated from foods and is only reduced by 1 or 2 logs, it may still render the food safer for consumption. For example, and to put this into a broader perspective, in 2003 the FDA and USDA’s FSIS jointly authored a risk-assessment study in which they modeled a series of “what if” scenarios, including one in which reductions in deli meat contamination would affect the mortality rate of elderly people. According to that analysis, a 10-fold reduction (1 log) and 100-fold reduction (2 logs) in pre-retail contamination with *L. monocytogenes* would reduce the mortality rate by ca. 50% and 74%, respectively in that segment of population [85]. Thus, implementation of phage biocontrol protocols—even if they do not eradicate (i.e., totally eliminate) the targeted foodborne pathogens from foods but reduce them by 1–3 logs—may yield significant improvements in food safety and public health.

Another technical challenge is related to the way phage biocontrol is implemented. Phage biocontrol provides an effective tool for improving food safety, but it does not eliminate the need for safe food handling practices. For example, regrowth of bacteria was observed after phage treatment if the foods are stored at abuse temperatures [33,48,54]. Also, some planning is required to maintain optimal efficacy of phage biocontrol when combining bacteriophages with some other food safety interventions, such as using phages in conjunction with chemical sanitizers [59]. For example, a number of chemical sanitizers are capable of inactivating phages, and therefore, they must be applied separately to ensure that phages retain viability in order to achieve the largest reductions of bacteria [59]. In this context, some investigators have reported that combinations of bacteriophage and preservatives are less effective than either treatment alone [86]. However, when proper synergistic combinations of phage preparations with other sanitizers are identified, the efficacy of each could be improved. For example, in the presence of high organic loads, the efficacy of a levulinic acid produce wash was enhanced (by up to 2 logs) when the fruits and vegetables were pretreated with a bacteriophage preparation [34]. 

Finally, another application-related (and efficacy-affecting) technical challenge is the possible emergence of phage-resistant bacterial isolates. Researchers do recover bacteria resistant to phage treatments [62], and there is the concern that widespread use of this treatment may eventually select for phage-resistant bacteria. Phages utilize a variety of bacterial structures to initiate the invasion of bacterial cells, including surface polysaccharides and proteins, as well as the flagella [87,88,89]. The use of phage cocktails containing multiple, diverse phages (e.g., phages that use different receptors on the surface of bacteria) versus a single monophage may provide a mechanism to reduce the risk/likelihood of bacterial resistance. Also, the intervention strategy itself can play a key role in managing the emergence of phage-resistant mutants. For instance, applying phages at the end of the food processing cycle (e.g., when phages are sprayed onto food immediately before packaging) reduces the overall “selective pressure” in the environment as bacterial exposure to the phages is limited. Consequently, there is less risk of phage-resistant mutants emerging when compared to, for example, spraying chicken houses or similar complex environments with phages in an effort to reduce the contamination of livestock animals. Finally, if and when resistance does arise, phage cocktails could be modified to include phages targeting previously resistant bacteria; one example of such an approach was published previously and discussed elsewhere in this review [16]. 

#### 3.2.2. Customer Acceptance

In recent years, consumers have increasingly demonstrated a reluctance to purchase foods treated with chemical sanitizers and antibiotics or foods that are “genetically modified”, while simultaneously the demand for organic foods and products produced locally, such as at local farmer’s markets and Community Supported Agriculture (CSA), has been on the rise [90,91]. This trend bodes well for phage biocontrol, which offers a non-chemical, green, and targeted antimicrobial approach for improving the safety of foods. However, the public may be disinclined to purchase foods processed with unfamiliar techniques, and the idea of “spraying viruses onto their food” could cause discomfort. Furthermore, food producers are generally reluctant to modify their practices, especially if there is a chance the public will react negatively. Thus, for phage biocontrol to be more widely utilized, it will be critical to provide education to the public and food processors, to explain the safety, efficacy, and ubiquity of bacteriophages. 

Phages are the most abundant organisms on the planet with approximately 10^31^ particles existing (ten times that of the total global bacterial population) [92], and approximately 10^15^ phage particles populating the human intestinal tract [93]. Phages are part of the normal microflora of all fresh foods [94], and they have been isolated from a variety of foods, from fruits and vegetables to meat and dairy products, often in very high numbers, e.g., up to 1 × 10^9^ PFU/mL in yogurt [95,96]. Phage biocontrol is also likely to be one of the most environmentally-friendly interventions available. In a previous review [18], we estimated that if phages were applied at the *maximum* approved amount (10^9^ PFU/g for one phage product, all other current approvals are for up to 10^7^–10^8^ PFU/g) to *all* the approved food consumed by the average American in one day, the phages consumed would represent <0.2% of the number of phages already present in the human intestinal tract. This calculation is a gross overestimate, especially considering several GRAS approvals permit an application of up to 10^8^ PFU/g (reducing the daily intake of phage to ~0.02% of the phage in the human intestinal tract). Also, this estimate assumes that (1) all possible food is treated, (2) all the applied phages survive the stomach acid and make it into the small intestine (yet most of the phages are usually destroyed when exposed to the acidic pH of the stomach), (3) the maximum approved amount of phages is applied, and (4) bacteriophage biocontrol is universally used by all relevant food industries in the United States. In short, the number of phages added to the environment and introduced into the human intestinal tract as a result of phage biocontrol is negligible, especially when compared to naturally present phage populations. Moreover, the phages in all currently available commercial products (Table 2) are not genetically modified and originated from the environment, potentially even from foods, in the first place. However, the general public is often not aware of these facts. Thus, proper understanding of the safe nature and ubiquity of lytic phages and the *pros* and *cons* of phage biocontrol by consumers and food processors alike will be critical for further successful implementation of this promising approach. In at least one recent study, consumers appeared to be willing to pay more for bacteriophage-treated fresh produce after the science behind phage biocontrol and the advantages of this technique were explained to them [97].

## 4. Concluding Remarks

Though some challenges remain, bacteriophage biocontrol is increasingly accepted as a safe and effective method to eliminate, or significantly reduce the levels of, specific bacterial pathogens from foods. Commercial bacteriophage products are currently available and have been approved for use in a growing number of countries. These products can be used to address contamination by specific bacterial pathogens at a variety of timepoints during food production, including spraying on produce, applying to livestock animals before processing, rinsing of food contact surfaces in processing facilities, and treatment of post-harvest food products, including RTE foods. Despite the progress made in improving the safety of our foods, foodborne illnesses remain a constant threat, especially for individuals with weaker immune systems, e.g., children, the elderly, and pregnant women. Bacteriophage biocontrol can serve as an additional tool in a multi-hurdle approach to prevent foodborne pathogens from reaching consumers, and this method is especially promising under circumstances when food processors strive to preserve the natural, and often beneficial, microbial population of foods and to only remove the bacteria that may cause illness in humans.

## Figures and Tables

**Table 1:** A summary of studies of direct phage application onto a variety of foods.

Bacterium *	Phages	Notes	Ref.
*Bacillus cereus*	BCP1-1	*Bacillus cereus* counts decreased after treatment with a single phage in fermented soya bean paste without affecting *Bacillus subtilis*, a critical component of the fermentation process.	[20]
*Campylobacter jejuni*	Φ2	Counts of *Campylobacter* were reduced by ~1 log on the surface of chicken skin stored at 4 °C after the application of a single phage.	[21]
*Campylobacter jejuni; Salmonella* spp.	*C. jejuni* typing page 12673, P22, 29C; *Salmonella* typing phage 12	*C. jejuni* levels decreased ~2 logs on experimentally-contaminated chicken skin after application of phage at a MOI of 100:1 or 1000:1. *Salmonella* levels were reduced by ~2 logs on chicken skin treated with phage at an MOI of either 100:1 or 1000:1 and stored for 48 h; bacterial counts were reduced below the limit of detection when lower levels of bacteria were used to contaminate the chicken.	[22]
*Campylobacter jejuni; Salmonella* spp.	Cj6; P7	*Campylobacter* levels significantly decreased in beef after application of the phage Cj6, and decreases in bacterial levels were not significant at low levels of bacterial contamination (~100 CFU/cm^2^). *Salmonella* counts were decreased ~2–3 logs at 5 °C and >5.9 logs at 24 °C in raw and cooked beef after P7 phage application. Surviving *Salmonella* colonies were still sensitive to P7. For both phages, the killing of bacteria was higher at an MOI of 10,000:1 and ~10,000 CFU/cm^2^ of bacteria.	[23]
*Cronobacter sakazakii*	ESP 1-3, ESP 732-1	In infant formula, *Cronobacter sakazakii* (formerly *Enterobacter sakazakii*) levels were decreased after phage addition. The reduction was dependent on the phage concentration, and the phages were more effective at 24 °C than 37 °C or 12 °C.	[24]
*Cronobacter sakazakii*	Five phages	Growth of 36 of 40 test strains was inhibited by a phage cocktail tested in infant formula experimentally contaminated with *C. sakazakii*. Furthermore, both high and low concentrations (10^6^ and 10^2^ CFU/mL) of bacteria were eliminated from liquid culture medium treated with the individual phage (10^8^ PFU/mL).	[25]
*Escherichia coli* O157:H7	e11/2, pp01, e4/1c	After incubation at 37 °C, a three-phage cocktail used to treat the surface of beef that was contaminated (10^3^ CFU/g) with *E. coli* O157:H7 eliminated the bacterium from a majority of the treated specimens.	[26]
*Escherichia coli* O157:H7	EcoShield™ (formerly ECP-100)	*E. coli* 0157:H7 levels decreased by ~1–3 logs, or were reduced below the limits of detection, on tomatoes, broccoli or spinach after treatment with a phage cocktail, while *E. coli* O157:H7 levels were decreased by ~1 log when the phages were applied to ground beef.	[17]
*Escherichia coli* O157:H7	EcoShield™ (formerly ECP-100)	A phage cocktail applied to experimentally contaminated lettuce and cut cantaloupe significantly reduced *E. coli* O157:H7 levels by up to 1.9 and 2.5 logs, respectively.	[27]
*Escherichia coli* O157:H7	Cocktail BEC8	At various temperatures (4, 8, 23 and 37 °C), the phage cocktail significantly reduced the level of *E. coli* O157:H7 on leafy green vegetables by ~2–4 logs. The inclusion of an essential oil (*trans*-cinnamaldehyde) increased this effect.	[28]
*Escherichia coli* O157:H7	EcoShield™ (formerly ECP-100)	The levels of *E. coli* O157:H7 were reduced by ≥94% and ~87% on the surface of experimentally contaminated beef and lettuce, respectively, after addition of the phage cocktail; however, the single treatment did not protect foods after recontamination with the same bacteria (i.e., phage biocontrol had no continued technical effect on the foods).	[29]
*Escherichia coli* O157:H7	EcoShield™ (formerly ECP-100)	After a 30 min phage treatment at both 4 and 10 °C, levels of *E. coli* O157:H7 decreased by >2 logs on leafy greens under both ambient and modified atmosphere packaging storage.	[30]
*Escherichia coli*	FAHEc1	Contamination of raw and cooked beef decreased by 2–4 logs at 5, 24 and 37 °C in a concentration dependent manner after phage application. The *E. coli* displayed regrowth at higher temperatures.	[31]
*Escherichia coli* O157:H7	EcoShield™ (formerly ECP-100)	A phage cocktail was applied to lettuce by spraying and dipping. A larger initial reduction (~0.8–1.3 logs) in *E. coli* O157:H7 counts was observed after spraying. Dipping required submerging the lettuce for as long as 2 min, and the initial reductions were not significant. After 1 day of storage at 4 °C, dipping in the highest concentration of the phage cocktail reduced *E. coli* by ~0.7 log.	[32]
*Escherichia coli*	EC6, EC9, EC11	Two *E. coli* strains were eradicated from raw and UHT milk after treatment with a three-phage cocktail at 5–9 °C and 25 °C. For a third *E. coli* strain, phage treatment eliminated the bacteria from UHT milk; however, after an initial reduction, regrowth occurred in the raw milk after 144 or 9 h for 5–9 °C and 25 °C storage, respectively.	[33]
*Escherichia coli, Salmonella, Shigella*	EcoShield™ (formerly ECP-100), SalmoFresh™, ShigActive™	Phage cocktails were as effective or more effective than chlorine wash at reducing targeted pathogenic bacteria from broccoli, cantaloupe and strawberries in samples containing a large amount of organic content. Combination of the phage cocktail and a produce wash generated a synergistic effect, i.e., higher reductions of bacteria.	[34]
*Listeria monocytogenes*	ListShield™ (formerly LMP-102)	*Listeria* counts decreased by ~2 logs and ~0.4 log after application of a phage cocktail on melon and apple slices, respectively; a synergistic effect was observed when phage and nisin were used, decreasing levels of *Listeria* on the fruit ~5.7 logs and ~2.3 logs, respectively.	[35]
*Listeria monocytogenes*	ListShield™ (formerly LMP-102)	Application of a phage cocktail 1, 0.5 or 0 h before honeydew melon tissue were contaminated with the bacterium was most effective at reducing *Listeria* counts. This effect depended on the concentration of phage applied. *Listeria* counts decreased by ~5–7 logs after 7 days, when the phages were applied at the times described above.	[36]
*Listeria monocytogenes*	PhageGuard Listex™ (formerly Listex™; P100)	Levels of *Listeria* were reduced by at least 3.5 logs after a single phage was administered to the surface of ripened red-smear soft cheese. The surviving *Listeria* colonies isolated from the cheese after phage treatment were not resistant to the phage.	[37]
*Listeria monocytogenes*	A511, PhageGuard Listex™ (formerly Listex™; P100)	Levels of *Listeria* in experimentally contaminated chocolate milk and mozzarella cheese brine were eradicated after phage treatment at 6 °C. When the phage cocktail was applied to various solid foods, including sliced cabbage, iceberg lettuce leaves, smoked salmon, mixed seafood, hot dogs, and sliced turkey meat, a reduction of *Listeria* of up to 5 logs was observed.	[38]
*Listeria monocytogenes*	PhageGuard Listex™ (formerly Listex™; P100)	*Listeria* counts decreased by 1.8–3.5 logs after application of a single phage at ~10^8^ PFU/g to the surface of raw salmon fillets that were stored at 4 °C or 22 °C.	[39]
*Listeria monocytogenes*	PhageGuard Listex™ (formerly Listex™; P100)	Levels of *Listeria* decreased by 1.4–2.0 logs CFU/g at 4 °C, 1.7–2.1 logs CFU/g at 10 °C, and 1.6–2.3 logs CFU/g at room temperature (22 °C) after application a single phage to the surface of raw catfish fillets. Regrowth was not observed after ten days of storage at either 4 °C or 10 °C.	[40]
*Listeria monocytogenes*	A511	The natural microbial community on soft cheese was maintained after addition of the phage. Levels of *Listeria* on experimentally contaminated cheese decreased by 2 logs and additional phage administrations did not improve the reduction of *Listeria*.	[41]
*Listeria monocytogenes*	FWLLm1	*Listeria* levels decreased by 1–2 logs on the surface of experimentally contaminated chicken stored in vacuum packages at 4 °C or 30 °C. Subsequent regrowth of *Listeria* was observed at 30 °C, but not at 4 °C.	[42]
*Listeria monocytogenes*	PhageGuard Listex™ (formerly Listex™; P100)	Counts of *Listeria* decreased by ~3 logs in experimentally contaminated queso fresco cheese after the addition of a single phage; however, subsequent growth was observed. Regrowth was prevented, and a similar log reduction was observed when PL + SD were included with the phage. Reduction of *Listeria* was lower, and regrowth occurred when LAE was included with phage.	[43]
*Listeria monocytogenes*	PhageGuard Listex™ (formerly Listex™; P100)	Compared to PL or PL + SD, a single phage was most effective at decreasing *Listeria* levels on RTE roast beef and turkey after storage at 4 °C or 10 °C, and subsequent bacterial growth was observed at both temperatures. Similar log reductions occurred when PL or PL + SD were used in conjunction with the phage, and regrowth was prevented or diminished at both 4 °C and 10 °C.	[44]
*Listeria monocytogenes*	PhageGuard Listex™ (formerly Listex™; P100)	Counts of *Listeria* decreased by ~1.5 logs on experimentally contaminated melon and pear slices, but not apple slices after two days at 10 °C. Additionally, treatment with phage did not impact *Listeria* levels in apple juice but decreased bacterial contamination by ~4 and ~2.5 logs in melon and pear juice, respectively.	[45]
*Listeria monocytogenes*	PhageGuard Listex™ (formerly Listex™; P100)	*Listeria* levels on soft cheese decreased by ~2–3 logs after 30 min and ~0.8–1 log after storage for 7 days at 10 °C.	[46]
*Listeria monocytogenes*	ListShield™ (formerly LMP-102)	Counts of *Listeria* decreased by 0.7 and 1.1 log on experimentally contaminated cheese and lettuce, respectively, after a 5 min treatment with phage and decreased the bacteria 1.1 log on the surface of apple slices after 24 h when combined with an antibrowning solution. The phage cocktail also virtually eliminated *Listeria* from experimentally contaminated frozen entrees that were frozen and thawed after treatment and was effective at eliminating environmental contamination by *Listeria* at a smoked salmon preparation plant.	[10]
*Listeria monocytogenes*	PhageGuard Listex™ (formerly Listex™; P100)	When applied to the surface of experimentally contaminated sliced pork ham, the phage reduced *Listeria* counts below the limit of detection after 72 h, and performed better than nisin, sodium lactate, or combinations of these antibacterial measures.	[47]
*Mycobacterium smegmatis*	Six phages	*M. smegmatis* counts were reduced below the limit of detection in milk treated with a six-phage cocktail or each component phage. Subsequent bacterial growth occurred when the component phages were used, but no growth was observed after 96 h at 37 °C, when the cocktail was applied.	[48]
*Salmonella* spp.	SJ2	*Salmonella* levels were reduced by 1–2 logs in raw and pasteurized cheeses created using milk that was treated with phage, while cheese made from milk without phage saw *Salmonella* counts rise ~1 log.	[49]
*Salmonella* spp.	SCPLX-1	Counts of *Salmonella* decreased by ~3.5 logs at 5 and 10 °C and ~2.5 logs at 20 °C on melon slices after application of a four-phage cocktail; treatment of apple slices with phage showed no reduction of bacteria.	[50]
*Salmonella* spp.	Felix-O1	*Salmonella* counts decreased by 1.8–2.1 logs after phage application to chicken frankfurters.	[51]
*Salmonella* spp.	PHL4	The levels of *Salmonella* recovered from experimentally contaminated broiler and naturally contaminated turkey carcasses were reduced by as high as 100% or 60%, respectively, after phage administration.	[52]
*Salmonella* spp.		Levels of *Salmonella* decreased by ~3 logs after application of a phage cocktail to sprouts; addition of an antagonistic bacteria to the phage cocktail increased this reduction to ~6 logs.	[53]
*Salmonella* spp.	FO1-E2	ln chocolate milk and mixed seafood, *Salmonella* levels were reduced to undetectable levels after phage treatment and storage for 24 h at 8 °C and remained below the limit of detection. When foods were phage-treated and stored at 15 °C, *Salmonella* counts were reduced to undetectable levels within 24–48 h for hot dogs, sliced turkey breast, and chocolate milk, but regrowth occurred after 5 days. *Salmonella* levels were initially inhibited at ~0.5–2 logs and ~1–3 logs in egg yolk and mixed seafood, respectively, after phage addition; but bacterial recovery matched controls in egg yolks after two days, while the log reduction was maintained in seafood.	[54]
*Salmonella* spp.	UAB_Phi 20, UAB_Phi78, UAB_Phi87	*Salmonella* counts decreased by ~1 log on the shells of fresh eggs and by 2–4 logs on lettuce 60 min after application of the phage. After an initial reduction of 1–2 logs, when chicken breasts were dipped in a phage cocktail, no further decrease in the bacterial counts was observed over the next seven days at 4 °C. The levels of *Salmonella* were reduced by 2–4 logs on pig skin after phage application and storage for 6 h at 33 °C.	[55]
*Salmonella* spp.	wksl3	*Salmonella* counts decreased by ~3 logs on chicken skin after application of a single phage, and no further decrease in bacterial levels was observed over the next seven days at 8 °C. Further, mice that received a single dose of phage orally displayed no adverse effects.	[56]
*Salmonella* spp.	Five phages	The levels of *Salmonella* decreased by ~1 log on chicken skin after application of a five-phage cocktail comprised of closely related phages. The reduction of bacteria achieved by the phages was comparable to three different chemical antimicrobials.	[57]
*Salmonella* spp.	P22	After the administration of a single temperate phage and storage at 4 °C, levels of *Salmonella* decreased by ~0.5–2 logs on chicken, below the limits of detection in whole and skimmed milk, ~3 logs in apple juice, ~2 logs in liquid egg, and ~2 logs in an energy drink.	[58]
*Salmonella* spp.	SalmoFresh™	The stability of a *Salmonella*-specific phage preparation was determined in various chemical antimicrobials. Treatment of chicken breast fillets with a combination of phages and individual chemical antimicrobials did not produce a synergistic effect on the reduction of *Salmonella*; however, application of chlorine or PAA followed by spraying with phages significantly reduced *Salmonella* from chicken skin by up to 2.5 logs, compared to use of chlorine, low levels of PAA, or phage alone (0.5–1.5 logs).	[59]
*Salmonella* spp.	SalmoFresh™	Treatment of chicken breast fillets by dipping or surface application of a *Salmonella*-specific bacteriophage preparation and storage at 4 °C significantly reduced *Salmonella* contamination by up to 0.9 log; further, storing the meat in modified atmospheric packaging after surface application produced a higher reduction in bacterial counts (up to 1.2 logs).	[60]
*Salmonella* spp.	SalmoLyse^®^	A phage cocktail was sprayed onto experimentally contaminated raw pet food ingredients, including chicken, tuna, turkey, cantaloupe, and lettuce, and reduced the levels of the targeted bacteria by ~0.4–1.1 logs.	[61]
*Salmonella* spp.	SJ2	Application of the phage SJ2 significantly reduced *Salmonella* colonies recovered from experimentally contaminated ground pork and eggs with a larger reduction observed at room temperature, compared to 4 °C. After treatment, *Salmonella* colonies were screened for phage resistance, and significantly more phage-resistant *Salmonella* isolates were recovered from eggs, compared with ground pork.	[62]
*Salmonella* spp.	PhageGuard S™ (formerly Salmonelex™)	Boneless chicken thighs and legs were experimentally contaminated with *Salmonella* serovars isolated from ground chicken or other sources. A larger reduction of *Salmonella* was achieved when the bacteriophage preparation was diluted in tap water, compared to filtered water prior to application, and the phage cocktail was more effective against *Salmonella* isolated from other sources, compared to those from ground chicken.	[63]
*Salmonella* spp.	PhageGuard S™ (formerly Salmonelex™)	Treatment with a bacteriophage cocktail or irradiation significantly reduced (~1 log) the level of *Salmonella* on experimentally contaminated ground beef trim, and a combination of these methods decreased bacterial contamination by ~2 logs.	[64]
*Shigella* spp.	SD-11, SF-A2, SS-92	*Shigella* counts were reduced by ~1–4 logs on pieces of spiced chicken after application of a phage cocktail or each of the component phages and storage at 4 °C.	[65]
*Shigella sonnei*	ShigaShield™	Application of a five-phage, *Shigella*-specific cocktail to various RTE foods, including lettuce, melon, smoked salmon, corned beef and pre-cooked chicken, reduced the recovery of *Shigella* ~1.0–1.4 logs at the highest phage concentration applied compared to control.	[66]
*Staphylococcus aureus*	Φ88, Φ35	*S. aureus* levels decreased below the limit of detection in experimentally contaminated whole milk after treatment, with a two-phage cocktail and storage at 37 °C. After phage treatment, *S. aureus* was not recovered from the acid curd after storage for 4 h at 25 °C, and was eliminated from the renneted curd after 1 h at 30 °C.	[67]
*Staphylococcus aureus*	vB_SauS-phi-IPLA35, vB_SauS-phi-SauS-IPLA88	Counts of *S. aureus* were significantly decreased in cheese made using milk treated with phages compared to milk without the addition of phages. The microbiota of the cheese was not impacted by the addition of the phages.	[68]

* Listed in alphabetical order by bacteria and then chronologically. In cases where multiple bacteria were examined, the study is listed with the alphabetically first bacteria. Adapted and modified from Sulakvelidze 2013 and Woolston and Sulakvelidze 2015 [12,18]. h, hour; LAE, lauric arginate; log(s), logarithmic unit(s); min, minutes; PAA, peracetic acid; PL, potassium lactate; PL + SD, potassium lactate + sodium diacetate; RTE, Ready-To-Eat; UHT, ultra-high temperature.

**Table 2:** Phage products approved for food safety applications.

Company	Phage Product	Target Organism(s)	Regulatory	Certifications	References
FINK TEC GmbH (Hamm, Germany)	Secure Shield E1	*E. coli*	FDA, GRN 724 *pending as of 19 March 2018*		
Intralytix, Inc. (Baltimore, MD, USA)	Ecolicide^®^ (EcolicidePX™)	*E. coli* O157:H7	USDA, FSIS Directive 7120.1		
	EcoShield™	*E. coli* O157:H7	FDA, FCN 1018; Israel Ministry of Health; Health Canada	Kosher; Halal	[17,27,29,30,32,34]
	ListShield™	*L. monocytogenes*	FDA, 21 CFR 172.785; FDA, GRN 528; EPA Reg. No. 74234-1; Israel Ministry of Health; Health Canada	Kosher; Halal; OMRI	[10,35,36]
	SalmoFresh™	*Salmonella* spp.	FDA, GRN 435; USDA, FSIS Directive 7120.1; Israel Ministry of Health; Health Canada	Kosher; Halal; OMRI	[59,60]
	ShigaShield™ (ShigActive™)	*Shigella* spp.	FDA, GRN 672		[66,69]
Micreos Food Safety (Wageningen, Netherlands)	PhageGuard Listex™	*L. monocytogenes*	FDA, GRN 198/218; FSANZ; EFSA; Swiss BAG; Israel Ministry of Health; Health Canada	Kosher; Halal; OMRI; SKAL	[37,38,39,40,43,44,45,46,47]
	PhageGuard S™	*Salmonella* spp.	FDA, GRN 468; FSANZ; Swiss BAG; Israel Ministry of Health; Health Canada	Kosher; Halal; OMRI; SKAL	[63,64]
		*E. coli* O157:H7	FDA, GRN 757 *pending as of 19 March 2018*		
Passport Food Safety Solutions (West Des Moines, IA, USA)	Finalyse^®^	*E. coli* O157:H7	USDA, FSIS Directive 7120.1		
Phagelux (Shanghai, China)	AgriPhage™	*Xanthomonas campestris* pv. vesicatoria, *Pseudomonas syringae* pv. tomato	EPA Reg. No. 67986-1		
	SalmoPro^®^	*Salmonella* spp.	FDA, GRN 603		
		*Salmonella* spp.	FDA, GRN 752 *pending as of March 19, 2018*		

Adapted and modified from Woolston and Sulakvelidze 2015 [18]. This is not meant to be an exhaustive list of phage products or approvals and listings. Some of the information included in this table was obtained from company webpages and promotional material and has not been independently verified. BAG, Bundesamt für Gesundheit; CFR, Code of Federal Regulations; FSIS, Food Safety and Inspection Service; GRN, GRAS Notice.

## References

[1] Havelaar A.H., Kirk M.D., Torgerson P.R., Gibb H.J., Hald T., Lake R.J., Praet N., Bellinger D.C., de Silva N.R., Gargouri N. (2015). World Health Organization global estimates and regional comparisons of the burden of foodborne disease in 2010. PLoS Med..

[2] Scharff R.L. (2012). Economic burden from health losses due to foodborne illness in the United States. J. Food Prot..

[3] Wolbang C.M., Fitos J.L., Treeby M.T. (2008). The effect of high pressure processing on nutritional value and quality attributes of *Cucumis melo* L.. Innov. Food Sci. Emerg..

[4] Bajovic B., Bolumar T., Heinz V. (2012). Quality considerations with high pressure processing of fresh and value added meat products. Meat Sci..

[5] Suklim K., Flick G.J., Vichitphan K. (2014). Effects of gamma irradiation on the physical and sensory quality and inactivation of *Listeria monocytogenes* in blue swimming crab meat (*Portunas pelagicus*). Radiat. Phys. Chem..

[6] Wheeler T.L., Shackelford S.D., Koohmaraie M. (1999). Trained sensory panel and consumer evaluation of the effects of gamma irradiation on palatability of vacuum-packaged frozen ground beef patties. J. Anim. Sci..

[7] Beuchat L.R., Ryu J.H. (1997). Produce handling and processing practices. Emerg. Infect. Dis..

[8] Sohaib M., Anjum F.M., Arshad M.S., Rahman U.U. (2016). Postharvest intervention technologies for safety enhancement of meat and meat based products; a critical review. J. Food Sci. Technol..

[9] Sulakvelidze A., Alavidze Z., Morris J.G. (2001). Bacteriophage therapy. Antimicrob. Agents Chemother..

[10] Perera M.N., Abuladze T., Li M.R., Woolston J., Sulakvelidze A. (2015). Bacteriophage cocktail significantly reduces or eliminates *Listeria monocytogenes* contamination on lettuce, apples, cheese, smoked salmon and frozen foods. Food Microbiol..

[11] Viator C.L., Muth M.K., Brophy J.E. (2015). Costs of Food Safety Investments.

[12] Sulakvelidze A. (2013). Using lytic bacteriophages to eliminate or significantly reduce contamination of food by foodborne bacterial pathogens. J. Sci. Food Agric..

[13] Schmelcher M., Loessner M.J. (2016). Bacteriophage endolysins: Applications for food safety. Curr. Opin. Biotechnol..

[14] Greer G.G. (2005). Bacteriophage control of foodborne bacteria. J. Food Prot..

[15] Lone A., Anany H., Hakeem M., Aguis L., Avdjian A.C., Bouget M., Atashi A., Brovko L., Rochefort D., Griffiths M.W. (2016). Development of prototypes of bioactive packaging materials based on immobilized bacteriophages for control of growth of bacterial pathogens in foods. Int. J. Food Microbiol..

[16] Woolston J., Parks A.R., Abuladze T., Anderson B., Li M., Carter C., Hanna L.F., Heyse S., Charbonneau D., Sulakvelidze A. (2013). Bacteriophages lytic for *Salmonella* rapidly reduce *Salmonella* contamination on glass and stainless steel surfaces. Bacteriophage.

[17] Abuladze T., Li M., Menetrez M.Y., Dean T., Senecal A., Sulakvelidze A. (2008). Bacteriophages reduce experimental contamination of hard surfaces, tomato, spinach, broccoli, and ground beef by *Escherichia coli* O157:H7. Appl. Environ. Microbiol..

[18] Woolston J., Sulakvelidze A., Chichester (2015). Bacteriophages and food safety. eLS.

[19] Endersen L., O’Mahony J., Hill C., Ross R.P., McAuliffe O., Coffey A. (2014). Phage therapy in the food industry. Annu. Rev. Food Sci. Technol..

[20] Bandara N., Jo J., Ryu S., Kim K.P. (2012). Bacteriophages BCP1-1 and BCP8-2 require divalent cations for efficient control of *Bacillus cereus* in fermented foods. Food Microbiol..

[21] Atterbury R.J., Connerton P.L., Dodd C.E., Rees C.E., Connerton I.F. (2003). Application of host-specific bacteriophages to the surface of chicken skin leads to a reduction in recovery of *Campylobacter jejuni*. Appl. Environ. Microbiol..

[22] Goode D., Allen V.M., Barrow P.A. (2003). Reduction of experimental *Salmonella* and *Campylobacter* contamination of chicken skin by application of lytic bacteriophages. Appl. Environ. Microbiol..

[23] Bigwood T., Hudson J.A., Billington C., Carey-Smith G.V., Heinemann J.A. (2008). Phage inactivation of foodborne pathogens on cooked and raw meat. Food Microbiol..

[24] Kim K.P., Klumpp J., Loessner M.J. (2007). *Enterobacter sakazakii* bacteriophages can prevent bacterial growth in reconstituted infant formula. Int. J. Food Microbiol..

[25] Zuber S., Boissin-Delaporte C., Michot L., Iversen C., Diep B., Brussow H., Breeuwer P. (2008). Decreasing *Enterobacter sakazakii* (*Cronobacter* spp.) food contamination level with bacteriophages: Prospects and problems. Microb. Biotechnol..

[26] O’Flynn G., Ross R.P., Fitzgerald G.F., Coffey A. (2004). Evaluation of a cocktail of three bacteriophages for biocontrol of *Escherichia coli* O157:H7. Appl. Environ. Microbiol..

[27] Sharma M., Patel J.R., Conway W.S., Ferguson S., Sulakvelidze A. (2009). Effectiveness of bacteriophages in reducing *Escherichia coli* O157:H7 on fresh-cut cantaloupes and lettuce. J. Food Prot..

[28] Viazis S., Akhtar M., Feirtag J., Diez-Gonzalez F. (2011). Reduction of *Escherichia coli* O157:H7 viability on leafy green vegetables by treatment with a bacteriophage mixture and *trans*-cinnamaldehyde. Food Microbiol..

[29] Carter C.D., Parks A., Abuladze T., Li M., Woolston J., Magnone J., Senecal A., Kropinski A.M., Sulakvelidze A. (2012). Bacteriophage cocktail significantly reduces *Escherichia coli* O157:H7 contamination of lettuce and beef, but does not protect against recontamination. Bacteriophage.

[30] Boyacioglu O., Sharma M., Sulakvelidze A., Goktepe I. (2013). Biocontrol of *Escherichia coli* O157:H7 on fresh-cut leafy greens. Bacteriophage.

[31] Hudson J.A., Billington C., Wilson T., On S.L. (2013). Effect of phage and host concentration on the inactivation of *Escherichia coli* O157:H7 on cooked and raw beef. Food Sci. Technol. Int..

[32] Ferguson S., Roberts C., Handy E., Sharma M. (2013). Lytic bacteriophages reduce *Escherichia coli* O157:H7 on fresh cut lettuce introduced through cross-contamination. Bacteriophage.

[33] McLean S.K., Dunn L.A., Palombo E.A. (2013). Phage inhibition of *Escherichia coli* in ultrahigh-temperature-treated and raw milk. Foodborne Pathog. Dis..

[34] Magnone J.P., Marek P.J., Sulakvelidze A., Senecal A.G. (2013). Additive approach for inactivation of *Escherichia coli* O157:H7, *Salmonella*, and *Shigella* spp. on contaminated fresh fruits and vegetables using bacteriophage cocktail and produce wash. J. Food Prot..

[35] Leverentz B., Conway W.S., Camp M.J., Janisiewicz W.J., Abuladze T., Yang M., Saftner R., Sulakvelidze A. (2003). Biocontrol of *Listeria monocytogenes* on fresh-cut produce by treatment with lytic bacteriophages and a bacteriocin. Appl. Environ. Microbiol..

[36] Leverentz B., Conway W.S., Janisiewicz W., Camp M.J. (2004). Optimizing concentration and timing of a phage spray application to reduce *Listeria monocytogenes* on honeydew melon tissue. J. Food Prot..

[37] Carlton R.M., Noordman W.H., Biswas B., de Meester E.D., Loessner M.J. (2005). Bacteriophage P100 for control of *Listeria monocytogenes* in foods: Genome sequence, bioinformatic analyses, oral toxicity study, and application. Regul. Toxicol. Pharmacol..

[38] Guenther S., Huwyler D., Richard S., Loessner M.J. (2009). Virulent bacteriophage for efficient biocontrol of *Listeria monocytogenes* in ready-to-eat foods. Appl. Environ. Microbiol..

[39] Soni K.A., Nannapaneni R. (2010). Bacteriophage significantly reduces *Listeria monocytogenes* on raw salmon fillet tissue. J. Food Prot..

[40] Soni K.A., Nannapaneni R., Hagens S. (2010). Reduction of *Listeria monocytogenes* on the surface of fresh channel catfish fillets by bacteriophage Listex P100. Foodborne Pathog. Dis..

[41] Guenther S., Loessner M.J. (2011). Bacteriophage biocontrol of *Listeria monocytogenes* on soft ripened white mold and red-smear cheeses. Bacteriophage.

[42] Bigot B., Lee W.J., McIntyre L., Wilson T., Hudson J.A., Billington C., Heinemann J.A. (2011). Control of *Listeria monocytogenes* growth in a ready-to-eat poultry product using a bacteriophage. Food Microbiol..

[43] Soni K.A., Desai M., Oladunjoye A., Skrobot F., Nannapaneni R. (2012). Reduction of *Listeria monocytogenes* in queso fresco cheese by a combination of listericidal and listeriostatic GRAS antimicrobials. Int. J. Food Microbiol..

[44] Chibeu A., Agius L., Gao A., Sabour P.M., Kropinski A.M., Balamurugan S. (2013). Efficacy of bacteriophage LISTEX^TM^ P100 combined with chemical antimicrobials in reducing *Listeria monocytogenes* in cooked turkey and roast beef. Int. J. Food Microbiol..

[45] Oliveira M., Viñas I., Colàs P., Anguera M., Usall J., Abadias M. (2014). Effectiveness of a bacteriophage in reducing *Listeria monocytogenes* on fresh-cut fruits and fruit juices. Food Microbiol..

[46] Silva E.N., Figueiredo A.C., Miranda F.A., de Castro Almeida R.C. (2014). Control of *Listeria monocytogenes* growth in soft cheeses by bacteriophage P100. Braz. J. Microbiol..

[47] Figueiredo A.C.L., Almeida R.C.C. (2017). Antibacterial efficacy of nisin, bacteriophage P100 and sodium lactate against *Listeria monocytogenes* in ready-to-eat sliced pork ham. Braz. J. Microbiol..

[48] Endersen L., Coffey A., Neve H., McAuliffe O., Ross R.P., O’Mahony J.M. (2013). Isolation and characterisation of six novel mycobacteriophages and investigation of their antimicrobial potential in milk. Int. Dairy J..

[49] Modi R., Hirvi Y., Hill A., Griffiths M.W. (2001). Effect of phage on survival of *Salmonella* Enteritidis during manufacture and storage of cheddar cheese made from raw and pasteurized milk. J. Food Prot..

[50] Leverentz B., Conway W.S., Alavidze Z., Janisiewicz W.J., Fuchs Y., Camp M.J., Chighladze E., Sulakvelidze A. (2001). Examination of bacteriophage as a biocontrol method for *Salmonella* on fresh-cut fruit: A model study. J. Food Prot..

[51] Whichard J.M., Sriranganathan N., Pierson F.W. (2003). Suppression of *Salmonella* growth by wild-type and large-plaque variants of bacteriophage Felix O1 in liquid culture and on chicken frankfurters. J. Food Prot..

[52] Higgins J.P., Higgins S.E., Guenther K.L., Huff W., Donoghue A.M., Donoghue D.J., Hargis B.M. (2005). Use of a specific bacteriophage treatment to reduce *Salmonella* in poultry products. Poult. Sci..

[53] Ye J., Kostrzynska M., Dunfield K., Warriner K. (2010). Control of *Salmonella* on sprouting mung bean and alfalfa seeds by using a biocontrol preparation based on antagonistic bacteria and lytic bacteriophages. J. Food Prot..

[54] Guenther S., Herzig O., Fieseler L., Klumpp J., Loessner M.J. (2012). Biocontrol of *Salmonella* Typhimurium in RTE foods with the virulent bacteriophage FO1-E2. Int. J. Food Microbiol..

[55] Spricigo D.A., Bardina C., Cortes P., Llagostera M. (2013). Use of a bacteriophage cocktail to control *Salmonella* in food and the food industry. Int. J. Food Microbiol..

[56] Kang H.W., Kim J.W., Jung T.S., Woo G.J. (2013). wksl3, a new biocontrol agent for *Salmonella enterica* serovars Enteritidis and Typhimurium in foods: Characterization, application, sequence analysis, and oral acute toxicity study. Appl. Environ. Microbiol..

[57] Hungaro H.M., Mendonça R.C.S., Gouvêa D.M., Vanetti M.C.D., Pinto C.L.D. (2013). Use of bacteriophages to reduce *Salmonella* in chicken skin in comparison with chemical agents. Food Res. Int..

[58] Zinno P., Devirgiliis C., Ercolini D., Ongeng D., Mauriello G. (2014). Bacteriophage P22 to challenge *Salmonella* in foods. Int. J. Food Microbiol..

[59] Sukumaran A.T., Nannapaneni R., Kiess A., Sharma C.S. (2015). Reduction of *Salmonella* on chicken meat and chicken skin by combined or sequential application of lytic bacteriophage with chemical antimicrobials. Int. J. Food Microbiol..

[60] Sukumaran A.T., Nannapaneni R., Kiess A., Sharma C.S. (2016). Reduction of *Salmonella* on chicken breast fillets stored under aerobic or modified atmosphere packaging by the application of lytic bacteriophage preparation SalmoFresh^TM^. Poult. Sci..

[61] Soffer N., Abuladze T., Woolston J., Li M., Hanna L.F., Heyse S., Charbonneau D., Sulakvelidze A. (2016). Bacteriophages safely reduce *Salmonella* contamination in pet food and raw pet food ingredients. Bacteriophage.

[62] Hong Y., Schmidt K., Marks D., Hatter S., Marshall A., Albino L., Ebner P. (2016). Treatment of *Salmonella*-contaminated eggs and pork with a broad-spectrum, single bacteriophage: Assessment of efficacy and resistance development. Foodborne Pathog. Dis..

[63] Grant A., Parveen S., Schwarz J., Hashem F., Vimini B. (2017). Reduction of *Salmonella* in ground chicken using a bacteriophage. Poult. Sci..

[64] Yeh Y., de Moura F.H., Van Den Broek K., de Mello A.S. (2018). Effect of ultraviolet light, organic acids, and bacteriophage on *Salmonella* populations in ground beef. Meat Sci..

[65] Zhang H., Wang R., Bao H.D. (2013). Phage inactivation of foodborne *Shigella* on ready-to-eat spiced chicken. Poult. Sci..

[66] Soffer N., Woolston J., Li M., Das C., Sulakvelidze A. (2017). Bacteriophage preparation lytic for *Shigella* significantly reduces *Shigella sonnei* contamination in various foods. PLoS ONE.

[67] Garcia P., Madera C., Martinez B., Rodriguez A. (2007). Biocontrol of *Staphylococcus aureus* in curd manufacturing processes using bacteriophages. Int. Dairy J..

[68] Bueno E., García P., Martínez B., Rodríguez A. (2012). Phage inactivation of *Staphylococcus aureus* in fresh and hard-type cheeses. Int. J. Food Microbiol..

[69] Mai V., Ukhanova M., Reinhard M.K., Li M., Sulakvelidze A. (2015). Bacteriophage administration significantly reduces *Shigella* colonization and shedding by *Shigella*-challenged mice without deleterious side effects and distortions in the gut microbiota. Bacteriophage.

[70] Tokman J.I., Kent D.J., Wiedmann M., Denes T. (2016). Temperature significantly affects the plaguing and adsorption efficiencies of *Listeria* phages. Front Microbiol..

[71] Hoskisson P.A., Smith M.C. (2007). Hypervariation and phase variation in the bacteriophage ‘resistome’. Curr. Opin. Microbiol..

[72] Freeman L.M., Chandler M.L., Hamper B.A., Weeth L.P. (2013). Current knowledge about the risks and benefits of raw meat-based diets for dogs and cats. J. Am. Vet. Med. Assoc..

[73] Behravesh C.B., Ferraro A., Deasy M., Dato V., Moll M., Sandt C., Rea N.K., Rickert R., Marriott C., Warren K. (2010). Human *Salmonella* infections linked to contaminated dry dog and cat food, 2006–2008. Pediatrics.

[74] Heyse S., Hanna L.F., Woolston J., Sulakvelidze A., Charbonneau D. (2015). Bacteriophage cocktail for biocontrol of *Salmonella* in dried pet food. J. Food Prot..

[75] Snyder A.B., Perry J.J., Yousef A.E. (2016). Developing and optimizing bacteriophage treatment to control enterohemorrhagic *Escherichia coli* on fresh produce. Int. J. Food Microbiol..

[76] Tomat D., Quiberoni A., Mercanti D., Balagué C. (2014). Hard surfaces decontamination of enteropathogenic and Shiga toxin-producing *Escherichia coli* using bacteriophages. Food Res. Int..

[77] Bower C.K., Daeschel M.A. (1999). Resistance responses of microorganisms in food environments. Int. J. Food Microbiol..

[78] Kotloff K.L., Winickoff J.P., Ivanoff B., Clemens J.D., Swerdlow D.L., Sansonetti P.J., Adak G.K., Levine M.M. (1999). Global burden of *Shigella* infections: Implications for vaccine development and implementation of control strategies. Bull. World Health Organ..

[79] Firlieyanti A.S., Connerton P.L., Connerton I.F. (2016). *Campylobacters* and their bacteriophages from chicken liver: The prospect for phage biocontrol. Int. J. Food Microbiol..

[80] Hammerl J.A., Jäckel C., Alter T., Janzcyk P., Stingl K., Knüver M.T., Hertwig S. (2014). Reduction of *Campylobacter jejuni* in broiler chicken by successive application of group II and group III phages. PLoS ONE.

[81] Zampara A., Sørensen M.C.H., Elsser-Gravesen A., Brøndsted L. (2017). Significance of phage-host interactions for biocontrol of *Campylobacter jejuni* in food. Food Control..

[82] Kittler S., Fischer S., Abdulmawjood A., Glunder G., Klein G. (2013). Effect of bacteriophage application on *Campylobacter jejuni* loads in commercial broiler flocks. Appl. Environ. Microbiol..

[83] Sorensen M.C., Gencay Y.E., Birk T., Baldvinsson S.B., Jackel C., Hammerl J.A., Vegge C.S., Neve H., Brondsted L. (2015). Primary isolation strain determines both phage type and receptors recognised by *Campylobacter jejuni* bacteriophages. PLoS ONE.

[84] Hudson J.A., McIntyre L., Billington C., Sabour P.M., Griffiths M.W. (2010). Application of bacteriophages to control pathogenic and spoilage bacteria in food processing and distribution. Bacteriophages in the Control of Food- and Waterborne Pathogens.

[85] US Food and Drug Administration Center for Food Safety and Applied Nutrition (FDA) (2003). Quantitative Assessment of Relative Risk to Public Health from Foodborne Listeria Monocytogenes Among Selected Categories of Ready-to-Eat Foods.

[86] Rodríguez E., Seguer J., Rocabayera X., Manresa A. (2004). Cellular effects of monohydrochloride of l-arginine, Nα-lauroyl ethylester (LAE) on exposure to *Salmonella typhimurium* and *Staphylococcus aureus*. J. Appl. Microbiol..

[87] Zhang H., Li L., Zhao Z., Peng D., Zhou X. (2016). Polar flagella rotation in *Vibrio parahaemolyticus* confers resistance to bacteriophage infection. Sci. Rep..

[88] Marti R., Zurfluh K., Hagens S., Pianezzi J., Klumpp J., Loessner M.J. (2013). Long tail fibres of the novel broad-host-range T-even bacteriophage S16 specifically recognize *Salmonella* OmpC. Mol. Microbiol..

[89] Lindberg A.A., Holme T. (1969). Influence of O side chains on the attachment of the Felix O-1 bacteriophage to *Salmonella* bacteria. J. Bacteriol..

[90] Reganold J.P., Wachter J.M. (2016). Organic agriculture in the twenty-first century. Nat. Plants.

[91] Woods T., Ernst M., Tropp D. (2017). Community Supported Agriculture–New Models for Changing Markets.

[92] Hatfull G.F. (2008). Bacteriophage genomics. Curr. Opin. Microbiol..

[93] Dalmasso M., Hill C., Ross R.P. (2014). Exploiting gut bacteriophages for human health. Trends Microbiol..

[94] Sulakvelidze A., Barrow P., Kutter E., Sulakvelidze A. (2005). Phage therapy in animals and agribusiness. Bacteriophages: Biology and Applications.

[95] Suárez V.B., Quiberoni A., Binetti A.G., Reinheimer J.A. (2002). Thermophilic lactic acid bacteria phages isolated from Argentinian dairy industries. J. Food Prot..

[96] Gautier M., Rouault A., Sommer P., Briandet R. (1995). Occurrence of *Propionibacterium freudenreichii* bacteriophages in swiss cheese. Appl. Environ. Microbiol..

[97] Naanwaab C., Yeboah O.A., Ofori Kyei F., Sulakvelidze A., Goktepe I. (2014). Evaluation of consumers’ perception and willingness to pay for bacteriophage treated fresh produce. Bacteriophage.

